# Application of Molecular Hydrogen in Heart Surgery under Cardiopulmonary Bypass

**DOI:** 10.17691/stm2021.13.1.09

**Published:** 2021-02-28

**Authors:** D.A. Danilova, Yu.D. Brichkin, A.P. Medvedev, V.V. Pichugin, S.A. Fedorov, E.V. Taranov, E.I. Nazarov, M.V. Ryazanov, G.V. Bolshukhin, A.V. Deryugina

**Affiliations:** Lecturer, Department of Physiology and Anatomy, Institute of Biology and Biomedicine, National Research Lobachevsky State University of Nizhni Novgorod, 23 Prospekt Gagarina, Nizhny Novgorod, 603950, Russia; Consulting Professor, Specialized Cardiosurgical Clinical Hospital named after Academician B.A. Korolev, 209 Vaneeva St., Nizhny Novgorod, 603136, Russia; Professor, Department of Hospital Surgery named after B.A. Korolev, Privolzhsky Research Medical University, 10/1 Minin and Pozharsky Square, Nizhny Novgorod, 603005, Russia; Professor, Department of Anesthesiology, Resuscitation and Emergency Medical Aid, Privolzhsky Research Medical University, 10/1 Minin and Pozharsky Square, Nizhny Novgorod, 603005, Russia; Cardiovascular Surgeon, Specialized Cardiosurgical Clinical Hospital named after Academician B.A. Korolev, 209 Vaneeva St., Nizhny Novgorod, 603136, Russia; Anesthesiologist-Resuscitator, Specialized Cardiosurgical Clinical Hospital named after Academician B.A. Korolev, 209 Vaneeva St., Nizhny Novgorod, 603136, Russia; Director, RPE “Ekonika”, 19 I. Rabina St., Odessa, 65078, Ukraine; Cardiovascular Surgeon, Specialized Cardiosurgical Clinical Hospital named after Academician B.A. Korolev, 209 Vaneeva St., Nizhny Novgorod, 603136, Russia; Cardiovascular Surgeon, Specialized Cardiosurgical Clinical Hospital named after Academician B.A. Korolev, 209 Vaneeva St., Nizhny Novgorod, 603136, Russia; Head of the Department of Physiology and Anatomy, Institute of Biology and Biomedicine, National Research Lobachevsky State University of Nizhni Novgorod, 23 Prospekt Gagarina, Nizhny Novgorod, 603950, Russia

**Keywords:** molecular hydrogen, lipid peroxidation, oxidative processes, antioxidants, cardiopulmonary bypass

## Abstract

**The aim of this work** was to study the effect of molecular hydrogen on oxidative processes in cardiac surgery patients with acquired valve heart disease applied during surgery under cardiopulmonary bypass (CPB).

**Materials and Methods.:**

The study involved 20 patients (16 men and 4 women) with acquired heart valve disease who were operated on under CPB. Two groups of patients were formed. In group 1 (n=11), anesthesia included inhalations of molecular hydrogen, which was supplied to the breathing circuit of the ventilator at a concentration of 1.5–2.0% immediately after tracheal intubation and throughout the operation. In group 2 (n=9), inhalation of molecular hydrogen was not performed. Blood sampling was taken at 4 stages: immediately after anesthesia induction, before CPB and after its termination, and also one day after the operation. The intensity of the processes of lipid peroxidation was evaluated by the level of diene (DC) and triene (TC) conjugates, Schiff bases (SB).

**Results.:**

In the patients of group 1, the arterial blood samples showed a decrease in the level of TC and SB, as compared to the first stage of the study, before the initiation of CPB and one day after the operation. An increase in the level of DC and TC was detected after the termination of CPB (p<0.05). In the venous blood samples, an increase in the level of DC was noted before the initiation of CPB, which was restored by the third stage of the study (p<0.05). At the same time, after the termination of CPB, a tendency towards a decrease in TC and SB was observed, which persisted one day after the operation.

In the patients of group 2, an increase in the concentration of SB in the arterial blood samples was recorded during the study as compared to the first stage. The level of TC and SB in the venous blood samples increased one day after the operation.

**Conclusion.:**

Intraoperative inhalation of molecular hydrogen leads to a decrease in the oxidative stress manifestation, it being most pronounced one day after the operation. This suggests that molecular hydrogen can be used in cardiac surgery as an effective and safe antioxidant.

## Introduction

In the modern world, in the countries with high and average living standards, diseases of the cardiovascular system are the leading cause of a decrease in the quality of life and mortality. Among them, a special place is occupied by acquired heart valve diseases that develop as a result of acute and chronic diseases and lead to a violation of intracardiac hemodynamics [1] which, in turn, can contribute to the development of circulatory failure in general [2]. The study of the pathology of the cardiovascular system is associated with the study of membrane disorders of cardiomyocytes which are caused by hypoxia, activation, or inhibition of enzyme systems. These disorders ultimately result in lipid peroxidation and the excess accumulation of toxic products of lipid peroxidation (LPO) [3] which are the cause of secondary damage to organs and tissues [4, 5]. Therefore, there is no doubt about the relevance of studying the LPO processes and their correction in cardiac surgery patients.

Numerous publications indicate that molecular hydrogen reduces oxidative stress both through direct reactions with strong oxidants [6] and indirectly, by regulating the expression of various genes having a versatile effect on the processes of inflammation [7], apoptosis [8], metabolism [9]. The therapeutic benefits of molecular hydrogen as compared to other known antioxidants are as follows: 1) a small size of the molecules allowing penetration through any biological membranes, including penetrating into mitochondria, where hydrogen suppresses cytotoxic free radicals at the site of their formation, and into the nucleus, where hydrogen prevents oxidative destruction of DNA; 2) selectivity of action: hydrogen neutralizes mainly cytotoxic radicals (•OH and ONOO^–^) without affecting less active signaling molecules [10]; 3) being a conventionally inert substance and having a zero redox potential in aqueous solutions, hydrogen does not exhibit toxic properties even after prolonged exposure in the form of highly concentrated gas mixtures [11].

The modern concepts suggest that the main predictor of the development of unfavorable adverse outcomes in the patients after cardiac surgery is the cytotoxic effect of reactive oxygen species (ROS) that is the oxidative stress due to the imbalance between the level of synthesized ROS and the ability of the redox system to level its growth. That is why the study of the role of oxidative stress in the pathogenesis of ischemic and reperfusion injuries in cardiac surgery and the search for methods for leveling these processes are highly relevant. The effect of molecular hydrogen on dynamics of oxidative processes during heart surgery has not been studied to date.

**The aim of this work** was to study the dynamics of oxidative parameters of blood plasma against the background of inhalation of molecular hydrogen in the process of anesthesia during the surgical correction of acquired heart valve disorders under cardiopulmonary bypass (CPB).

## Materials and Methods

The study included 20 patients with acquired heart valve disease (16 men and 4 women) who were operated on under cardiopulmonary bypass in the Specialized Cardiosurgical Clinical Hospital named after Academician B.A. Korolev. The mean age of the patients was 57.6±7.8 years.

The patients were divided into two groups. Group 1 consisted of 11 subjects, who were given anesthesia with inhalation of molecular hydrogen. The latter was obtained using a Bozon-H H_2_ hydrogen generator (RPE “Ekonika”, Ukraine) and supplied to the respiratory circuit of the ventilator at a concentration of 1.5–2.0% immediately after tracheal intubation and throughout the operation. Group 2 consisted of 9 patients who did not receive hydrogen inhalation. The severity of the patients’ condition was determined according to the classification of the New York Heart Association (NYHA). The overwhelming majority of patients in both groups were assigned to functional class III: 9 out of 11 subjects in group 1 and 6 out of 9 subjects in group 2.

The mean CPB time was 74.3±25.8 min in the patients of group 1 and 76.1±27.3 min in the patients of group 2. The mean time of aortic cross-clamping was 67.2±18.2 and 60.2±13.2 min, respectively (Table 1).

**Table 1:** Clinical features of the patients (M±σ)

Features	Group 1	Group 2
Age (years)	57.1±6.3	62.3±5.5
Sex (male/female)	2/9	2/7
CPB duration (min)	74.3±25.8	76.1±27.3
Duration of aortic cross-clamping (min)	67.2±18.2	60.2±13.2

The groups did not have significant differences in age and sex, severity of the condition, the nature of the surgical intervention, CPB duration, and aortic cross-clamp time. All the patients received standard premedication with diazepam (0.15 mg/kg) intramuscularly 30 min before surgery. Anesthesia was induced by a combination of diazepam (0.2–0.3 mg/kg) and propofol (2 mg/kg). Anesthesia was maintained at all stages of the operation using sevoflurane, an inhalational anesthetic. Fentanyl was used as an additional analgesic component. Myoplegia was maintained with Arduan at a dose of 0.1 mg/kg.

The study was approved by the Ethics Committee of the Specialized Cardiosurgical Clinical Hospital named after Academician B.A. Korolev (Nizhny Novgorod, Russia), in compliance with the World Medical Association Declaration of Helsinki “Ethical principles for medical research involving human subjects” and the requirements stated in the main regulatory documents of the Russian Federation on clinical trials. Written informed consent was obtained from all the patients.

Blood sampling was taken at 4 stages of the operation: 1^st^ — immediately after the induction of anesthesia; 2^nd^ — before CPB initiation; 3^rd^ — after the termination of CPB, 4^th^ — one day after the operation. We studied the intensity of LPO processes by the contents of diene (DC) and triene (TC) conjugates, Schiff bases (SB) in blood plasma, which were determined by the method of I.A. Volchegorsky (1989). A heptane-isopropanol mixture (1:1) was added to the plasma, shaken, then 1 ml of an aqueous solution of hydrochloric acid (pH 2), and 2 ml of heptane were added. After settling and stratification into phases of the resulting mixture, the optical densities (E) were measured on an SF-2000 spectrophotometer (Spectr, Russia), evaluating each phase at wavelengths of 220 nm (absorption of broken double bonds), 232 nm (absorption of DC), 278 nm (absorption of TC), 400 nm (absorption of SB).

The obtained data were processed using the BIOSTAT (AnalystSoft Inc., USA) and Microsoft Excel (Microsoft, USA) software packages using one-dimensional statistical models. The Shapiro–Wilk test was used to check the hypothesis about the type of distribution. The statistical patterns in the samples were studied with the parametric Student’s t-test since a normal distribution was shown. The parameters such as the arithmetic mean of the sample population and the standard deviation were calculated according to the Student’s t-test. The differences were considered significant at a significance level of p<0.05.

## Results and Discussion

**Figure 1:**
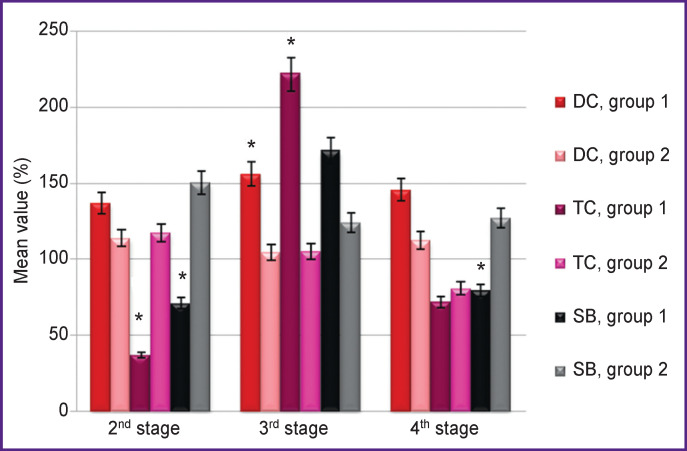
Dynamics of the levels of diene, triene conjugates, and Schiff bases in arterial blood plasma (as a percentage of the values before surgery) Here: 100% — values at the 1^st^ stage (after anesthesia induction); * statistically significant differences in the values of group 1 relative to the comparison group at the research stages (p<0.05)

In the course of the study it was revealed that when molecular hydrogen was used in arterial blood samples before the initiation of the cardiopulmonary bypass procedure and one day after the operation, a decrease in the levels of TC and SB as compared to stage 1 was observed, although the levels of DC and TC increased after the termination of cardiopulmonary bypass (Figure 1, Table 2). In the comparison group, there was an increase in SB as compared to the 1^st^ stage, most significantly at the 2^nd^ stage, before CPB initiation.

**Table 2:** Dynamics of diene, triene conjugates, and Schiff bases in arterial blood samples of the studied groups (RU), (M±σ)

Stage of the study	Group 1			Group 2		
	DC	TC	SB	DC	ТC	SB
After anesthesia induction	1.365±0.511	2.027±0.333	3.261±0.568	1.413±0.566	0.768±0.227	0.840±0.291
Before CPB initiation	1.870±0.457	0.756±0.281^*^	2.325±0.692^*^	1.612±0.344	0.903±0.265	1.262±0.116^*^
On CPB termination	2.130±0.369^*^	4.492±1.007^*^	5.616±1.771	1.475±0.455	0.807±0.235	1.042±0.462
One day after the operation	1.987±0.764	1.459±0.479	2.597±0.396^*^	1.589±0.265	0.622±0.253	1.167±0.361

^*^ Statistically significant differences in values as compared to those before surgery (p<0.05).

It should be noted that SB are formed by the covalent bond formation between LPO secondary products and the N-terminal residues of amino acids, proteins, and amino groups of phospholipids, and they also enter into polymerization and polycondensation reactions. Then the functional properties of the membranes are lost in this case, their destabilization occurs that results in cell destruction [12].

**Figure 2:**
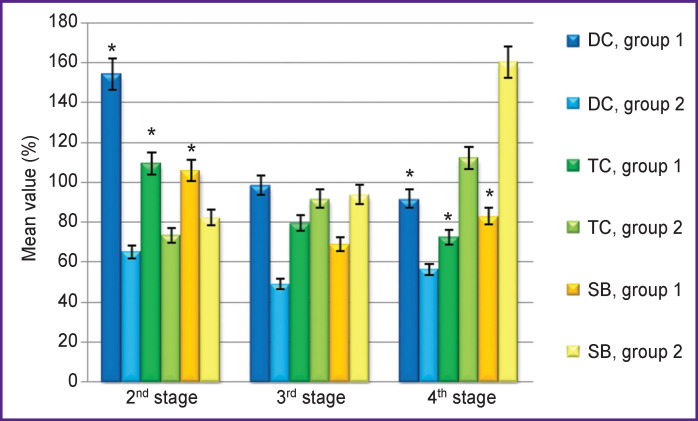
Dynamics of the levels of diene, triene conjugates, and Schiff bases in venous blood plasma (as a percentage of the values before surgery) Here: 100% — values at the 1^st^ stage (after anesthesia induction); * statistically significant differences in the values of group 1 as compared with the comparison group at the research stages (p<0.05)

The analysis of the dynamics of the venous blood showed that, at the application of hydrogen, despite an increase in the DC level at the 2^nd^ stage of the study, it decreases by the 3^rd^ stage, with subsequent decrease one day after the operation (Figure 2, Table 3). At the same time, after the termination of CPB (3^rd^ stage), a tendency to decrease in DC and SB was observed. This tendency persisted one day after the operation. In group 2, the level of TC and SB, on the contrary, increased one day after the operation, significant changes occurring in SB on the first day after the operation.

**Table 3:** Dynamics of diene, triene conjugates, and Schiff bases in the venous blood samples of the studied groups (RU), (M±σ)

Stage of the study	Group 1			Group 2		
	DC	TC	SB	DC	TC	SB
After anesthesia induction	1.797±0.454	0.906±0.569	1.171±0.493	3.152±0.544	1.165±0.879	1.032±0.242
Before CPB initiation	2.774±0.618	0.992±0.615	1.243±0.405	2.053±1.067	0.857±0.389	0.850±0.463
On CPB termination	1.773±0.905	0.720±0.294	0.810±0.352	1.545±0.375^*^	1.069±0.482	0.969±0.381
One day after the operation	1.648±0.438	0.657±0.407	0.972±0.525	1.775±0.845^*^	1.308±0.694	1.655±0.217^*^

^*^ Statistically significant differences in the values as compared to those before surgery (p<0.05).

The normal LPO intensity is known to meet the requirements of cellular metabolism, ensuring the vital activity of cells and their normal functioning. The excessive activation of these processes leads to membrane damage and is observed during the development of pathology [13]. The analysis of the data obtained in the course of the study suggests that the use of molecular hydrogen in heart valve surgery reduces the level of oxidative stress.

When discussing the mechanisms of hydrogen action, it should be mentioned that there is a possibility of direct reduction of hydroxyl radicals (•OH) and peroxynitrite (ONOO^–^) with molecular hydrogen [14]. •OH-radical is known to be the main trigger of the chain reaction of free radicals [15]. Hydrogen can accumulate in the lipid phase, particularly in the unsaturated lipid regions which are the main target of the initial chain reaction, and suppress the reaction that results in the formation of lipid peroxide [16, 17]. It neutralizes •OH-radicals, while less active ROS that perform the function of signaling molecules and are necessary for normal metabolism are not affected [18, 19].

ONOO^–^ radical modifies the tyrosine of proteins to form nitrotyrosine. Several studies [14, 16] have shown that application of molecular hydrogen reduces effectively the level of nitrotyrosine in animal models despite the form of hydrogen delivery into the body. Hence, at least, part of the effect of hydrogen can be explained by a decrease in the production of nitrotyrosine by proteins. Many protein factors involved in the control of transcription are nitrolyzed (–O–NO_2_) or nitrosated (–S–NO_2_). Therefore, a decrease in –О– NO_2_ or –S–NO_2_ can regulate various gene expressions.

Moreover, hydrogen reduces oxidative stress not only directly but also indirectly stimulating the growth of components of the antioxidant system, including heme oxygenase-1 (HO-1), superoxide dismutase (SOD), catalase, and myeloperoxidase [20]. The erythroid p45-related nuclear factor 2 (Nrf2) is known to function as a defense system against oxidative stress by inducing various enzymes of the antioxidant response. HO-1 is a microsomal enzyme that breaks down heme (a precursor of hemoglobin) to carbon monoxide, free iron, and biliverdin, and is involved in cell protection from oxidative stress. Hydrogen-assisted activation of Nrf2 promotes enhanced antioxidant protection [16].

Thus, the results of the arterial and venous blood tests demonstrate that the concentration of LPO products in the postoperative period in the first (main) study group decreases due to the direct antioxidant effect of molecular hydrogen and possible indirect effect through the modulation of the concentration of reactive oxygen species, which are natural signaling messengers, and they are essential for the regulation of cell activity.

## Conclusion

The results obtained suggest that intraoperative inhalations of molecular hydrogen can be used in cardiac surgery as an effective and safe antioxidant.

## References

[1] Shlyahto E.V. (2015). Kardiologiya: natsional’noe rukovodstvo.

[2] Urazova G.E., Landyshev Yu.S., Vakhnenko Yu.V., Pogrebnaya M.V., Vymenkhin A.N. (2010). Priobretennye poroki serdtsa. Diagnostika i lechenie.

[3] Artykova T.K., Ismailov K.I (2015). Lipid peroxidation and antioxidant protection in children with vegetative-vascular dystonia.. Vestnik Avitsenny.

[4] Lankin V.Z., Tikhaze A.K., Belenkov Yu.N. (2000). Free radical processes in diseases of the cardiovascular system. Kardiologia.

[5] Okunevich I.V., Sapronov N.S (2004). Antioxidants: the effectiveness of natural and synthetic compounds in the complex therapy of cardiovascular diseases.. Obzory po kliniсeskoj farmakologii i lekarstvennoj terapii.

[6] Li J., Wang C., Zhang J., Cai J.M., Cao Y.P., Sun X.J (2010). Hydrogen-rich saline improves memory function in a rat model of amyloid-beta-induced Alzheimer’s disease by reduction of oxidative stress.. Brain Res.

[7] Xie K., Yu Y., Zhang Z., Liu W., Pei Yu., Xiong L., Hou L., Wang G. (2010). Hydrogen gas improves survival rate and organ damage in zymosan-induced generalized inflammation model. Shock.

[8] Yang Y., Li B., Liu C., Chuai Yu., Lei J., Gao F., Cui J., Sun D., Cheng Y., Zhou C., Cai J. (2012). Hydrogen-rich saline protects immunocytes from radiation-induced apoptosis. Med Sci Monit.

[9] Song G., Li M., Sang H., Zhang L., Li X., Yao S., Yu Y., Zong C., Xue Y., Qin S (2013). Hydrogen-rich water decreases serum LDL-cholesterol levels and improves HDL function in patients with potential metabolic syndrome.. J Lipid Res.

[10] Hong Y., Chen S., Zhang J.M (2010). Hydrogen as a selective antioxidant: a review of clinical and experimental studies.. J Int Med Res.

[11] Dixon B.J., Tang J., Zhang J.H (2013). The evolution of molecular hydrogen: a noteworthy potential therapy with clinical significance.. Med Gas Res.

[12] Tarasov N.I., Teplyakov A.T., Malakhovich E.V., Stepacheva T.A., Kalyuzhin V.V., Pushnikova E.Yu., Fedosova N.N. (2002). The state of lipid peroxidation, antioxidant protection of blood in patients with myocardial infarction, aggravated by circulatory insufficiency. Terapevticheskii arkhiv.

[13] Boyarinov G.A., Boyarinova L.V., Deryugina A.V., Solov’eva O.D., Zaytsev R.R., Voyennov O.V., Moshnina E.V., Shumilova A.V (2016). Role of secondary brain damage factors in activation of vascularplatelet hemostasis in traumatic brain injury.. Obsaa reanimatologia.

[14] Ge L., Yang M., Yang N.N., Yin X.X., Song W.G (2017). Molecular hydrogen: a preventive and therapeutic medical gas for various diseases.. Oncotarget.

[15] Deryugina A.V., Boyarinov G.A., Simutis I.S., Nikolskiy V.O., Kuznetsov A.B., Efimova T.S (2018). Сorrection of metabolic indicators of erythrocytes and the structure of myocardium after acute blood loss using an ozonized erythrocytal mass.. Tsitologiya.

[16] Ohta S (2014). Molecular hydrogen as preventive and therapeutic medical gas: initiation, development and potential of hydrogen medicine.. Pharmacol Ther.

[17] Boyarinov G.A., Simutis I.S., Nikolsky V.O., Deryugina A.V., Boyarinova L.V., Gordetsov A.S., Kuznetsov A.B (2018). The role of ozonized erythrocytic mass transfusion in the restoration of myocardial morphological changes during blood loss (experimental study).. Obsaa reanimatologia.

[18] Ohsawa I., Ishikawa M., Takahashi K., Watanabe M., Nishimaki K., Yamagata K., Katsura K., Katayama Y., Asoh S., Ohta S (2007). Hydrogen acts as a therapeutic antioxidant by selectively reducing cytotoxic oxygen radicals.. Nat Med.

[19] Chepur S.V., Pluzhnikov N.N., Khurtsilava O.G., Maevsky E.I., Gogolevsky A.S., Tyunin M.A., Bakulina L.S., Lobeeva A.S (2017). Biological effects of molecular hydrogen and its application in clinical practice.. Uspehi sovremennoj biologii.

[20] Li H.M., Shen L., Ge J.W., Zhang R.F. (2017). The transfer of hydrogen from inert gas to therapeutic gas. Med Gas Res.

